# The Differences of Remineralization Potential of Bioactive Resin Infiltration and Conventional Resin Infiltration on Artificial Demineralized Enamel

**DOI:** 10.1155/tswj/3585365

**Published:** 2026-05-07

**Authors:** Kemporn Kitsahawong, Apa Juntavee, Niwut Juntavee, Chalita Suwannasuk, Chayapha Siriwatthanamethanon, Nattanicha Buddeesee, Rachata Somsub

**Affiliations:** ^1^ Department of Preventive Dentistry, Faculty of Dentistry, Khon Kaen University, Khon Kaen, Thailand, kku.ac.th; ^2^ Department of Prosthodontics, Faculty of Dentistry, Khon Kaen University, Khon Kaen, Thailand, kku.ac.th; ^3^ Division of Biomaterials Research, Faculty of Dentistry, Khon Kaen University, Khon Kaen, Thailand, kku.ac.th

## Abstract

**Introduction:**

Management of demineralized enamel has focused on the use of materials for remineralization. This study compared the remineralization potential of bioactive remineralizing resin (2R) with conventional resin (CR) infiltration on demineralized enamel.

**Material and Methods:**

Sixty demineralized enamel premolars were generated, allocated for three groups for infiltration with 2R, CR, and no treatment (NT), and subjected to pH cycling. Samples were determined for microhardness of enamel at the levels 50–100–200–300 *μ*m from the external surface. Polarized light microscopy (PLM) and energy dispersive spectroscopy were assessed for infiltration depth and calcium/phosphorus ratio (Ca/P). ANOVA and Fisher′s test were analyzed for significant differences in microhardness and Ca/P among groups (*α* = 0.05).

**Results:**

2R exhibited significantly higher hardness and Ca/P to demineralized enamel than CR and NT for every depth (*p* < 0.05), except for the hardness of 2R versus CR at 50 and 100 *μ*m, and Ca/P of 2R‐100 and 200 *μ*m versus CR‐50 *μ*m. 2R showed significantly higher infiltration and remineralization to demineralized enamel than CR and NT. PLM denoted a greater decrease in depth of carious lesion for 2R, compared with CR, with no depth reduction for NT.

**Conclusion:**

2R is capable of penetrating and enhancing the hardness of demineralized enamel more than CR, compared with NT. Increasing Ca/P upon infiltration with 2R over CR and NT denoted the capability of remineralization to carious lesions. A decrease in the depth of carious lesions was observed upon 2R application, more than CR and NT.

**Clinical Relevance:**

2R infiltration demonstrated an effective remineralization material for handling initial carious lesions in dental practice.

## 1. Introduction

The management of dental caries has undergone considerable evolution in recent years. Treatment options for caries lesions now include minimal intervention, known as noninvasive treatment [1]. Resin infiltration is one of the materials used for minimally invasive dental procedures. The indications for resin infiltration are to treat initial dental caries or demineralization of smooth surfaces of the tooth, such as white spot lesions, moderate to mild fluorosis, hypoplasia, discoloration caused by acute trauma, and lesions in molar incisor hypomineralization [2]. The conventional resin (CR) infiltration material, which is a broadly commercialized product, utilizes resin for infiltration of the early stage of dental caries, particularly in the proximal areas and smooth surfaces [1]. Resin infiltration of proximal caries lesions is effective in significantly reducing lesion progression in clinical trials and halts the progression of demineralized lesions by occluding porosities and concealing lesions [1–3]. Nevertheless, it has no capability of remineralization on demineralized enamel. It is important to note that CR infiltration presents an artificial and rough surface that is prone to heavy colonization by cariogenic biofilms. Consequently, the lack of buffering capacity has been associated with elevated rates of secondary caries occurrence in the tooth structure adjacent to resin restorations [4].

Remineralized resin (2R) infiltration is a bioactive resin infiltration material that incorporates nanohydroxyapatite (nHA, Sangi, Tokyo, Japan) and Apacider (Apacider–AW, Sangi) to provide antibacterial effects through nanoparticles of silver and zinc in the Apacider as well as remineralization and antidemineralization potential through nHA [5, 6]. The 2R infiltration is the method that uses a resin‐based material to penetrate the porous demineralized enamel for stabilizing the lesion and preventing further progression. The inclusion of bioactive agents, such as nHA and Apacider, into base resin enhances the therapeutic potential by promoting remineralization and restoring enamel structure [5, 6]. The nHA is capable of mimicking the structure and composition of natural enamel crystalline apatite. It demonstrates high biocompatibility and has been approved for use as an anticaries agent [7, 8]. The nHA is a nano‐sized compound of calcium phosphate in the form of hydroxyapatite that closely resembles the shape and structure of the apatite crystals found in human tooth enamel. Hence, it can be used to substitute natural minerals in human enamel for biomimetic purposes [9]. It possesses excessive surface area and more capability to integrate into the mineral matrix of teeth, rendering excellent remineralization [10]. A previous study reported that the application of nHA gel was significantly capable of increasing enamel microhardness, reducing lesion depth, and promoting the formation of a characteristic layer of apatite [6]. Furthermore, nHA in the form of either toothpaste or gel is further effective in promoting remineralization than fluoride varnish [6]. The nHA particles with a size of 20–60 nm closely match the dimensions of nano‐defects on the enamel surface resulting from acidic erosion. These nanoparticles can adhere to demineralized enamel surfaces and effectively prevent further acid damage [11]. Incorporating nHA into 2R infiltration material provides several benefits. Firstly, the similarity of nHA to the natural tooth structure allows for highly effective remineralization, as the nanocrystalline structure can easily penetrate and fill the microscopic spaces within the demineralized tooth structure. Secondly, once nHA has been integrated into the tooth structure, it can contribute to the mechanical properties of the enamel by increasing the resistance to wear and acid attacks, promoting the reduction of bacterial biofilm formation, and strengthening tooth structure. Conclusively, it enhances the long‐term protective effect of 2R infiltration material. Apacider is a unique antibacterial material comprising silver, zinc, nano‐silica, and tricalcium phosphate. It is included in the 2R infiltration material for the purpose of providing remineralization and antibacterial properties [5, 6, 12]. It can release silver ions (Ag^+^) and zinc ions (Zn^2+^) that function as an effective antibacterial agent. These ions disrupt bacterial cell membranes and inhibit the metabolic processes of bacteria, especially *S. mutans,* which is a prime bacterium causing dental caries. They prevent bacterial biofilm formation and reduce acid production, which is crucial for caries prevention [12]. The nano‐silica and tricalcium phosphate enable remineralization of carious lesions [5, 6].

The combination of base resin, Apacider, and nHA in the 2R infiltration material offers a synergistic effect that goes beyond the benefits of each solitary component. The base resin provides a durable physical barrier, whereas Apacider offers an antibacterial effect, whereas nHA directly integrates into the tooth structure, restoring strength and aesthetics [5, 6, 12]. This multifaceted approach allows for both the arrest of caries progression and the regeneration of tooth structure, offering comprehensive treatment for initial carious lesions. Besides, 2R Infiltration is potentially considered for minimally invasive dentistry that allows dentists to treat lesions without drilling or removing significant amounts of healthy tooth structure. Patients benefit from painless, noninvasive treatment. Therefore, 2R infiltration would not only reduce carious lesions but also promote the natural healing of the tooth. The 2R Infiltration would be considered an advanced method in the management of initial carious lesions by combining the durable qualities of resin with the bioactive aspects of Apacider and the enamel‐like characteristics of nHA. This novel bioactive material may provide both preventative and restorative innovation in dentistry. The 2R infiltration material reflects a novel, unconventional approach to counteract early carious lesions sooner or later. To date, no published studies have directly compared the remineralization potential and infiltration performance of 2R and CR infiltration materials on artificial demineralized enamel lesions. Therefore, the present study is aimed at evaluating and comparing the remineralization effectiveness of 2R infiltration and CR infiltration materials on artificial enamel lesions. The null hypotheses were that no significant differences would be observed in remineralization potential or infiltration performance between the two materials when compared with untreated demineralized enamel.

## 2. Materials and Methods

The study was endorsed by the Khon Kaen University (KKU) ethics committee for research in humans (Reference No: HE682048) and tailed the CRIS standards for an in vitro study. The sample size was anticipated with the Pi‐face software program version 1.76 (Iowa University, Iowa, Iowa, United States) using the data from the previous study [13] for calculation in Equation (1) at a power of test = 0.90 and a 95% level of significance (*α* = 0.05).

(1) N per group=Zα/2+Zβ2 s12+s22μ1−μ22

Where *Z*_*α*_ is the normal standard deviation (SD) = 1.96 (*α* = 0.05), *Z*_*β*_ is the normal SD = 1.28 (*β* = 0.1), *μ*_1_−*μ*_2_ is the difference of mean between groups = 54.89, and *s* is the standard deviation (*s*_1_ = 49.03, *s*_2_ = 40.95). The sample size of 20 samples per group was used in this investigation.

All experimental procedures were performed by a single expert operator following blinded protocols.

### 2.1. Sample Preparation

**Figure 1:**
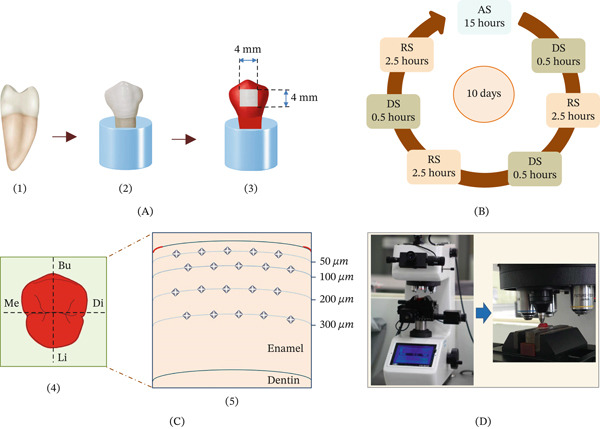
(A) A human bicuspid was selected (1) and embedded the root portion into the acrylic block (2). The coronal surface was coated with varnish except for the area of 4 × 4  mm^2^ on the buccal (Bu) and lingual (Li) surfaces (3). (B) The samples were treated in the cycling process comprising the demineralized solution (DS), remineralized solution (RS), and artificial saliva (AS) for 10 cycles. (C) The tooth sample was vertically sectioned in the bucco‐lingual and mesio‐distal direction (4), exposing the inner surface of enamel for determination of microhardness at 50, 100, 200, and 300 *μ*m apart from the external surface of enamel (5) by using (D) a Vickers diamond indenter in the microhardness testing apparatus.

Sixty extracted human premolars indicated for orthodontic purposes were collected from the dental hospital of Khon Kaen University. Teeth with developmental defects, enamel hypoplasia, fluorosis, white spot lesions, cracks, abrasions, or caries were excluded. The patient and guardian were advised and signed the consent form before the tooth extraction. The removed teeth were conserved in the 0.1% thymol solution (M‐Dent, Bangkok, Thailand) until starting the experimentation. All teeth were cleansed of any plaque, calculus, or soft tissue remnants and washed out with deionized (DI) water before being used in the experiment (Figure 1(A1)). The root portion of the tooth was fixed in the resin block, and the coronal portion and the cementoenamel junction (CEJ) were left over the resin (Figure 1(A2)). The tooth surfaces and resin block were painted with the fingernail varnish (Jessie Milano, Arluno, Milan, Italy) except for the 4 × 4 mm of tested area in the midmost part of the buccal and lingual surface of enamel, which was left uncoated (Figure 1(A3)).

### 2.2. Induction of Artificial Demineralized Enamel Lesion

The formation of an artificial demineralized enamel was performed by using a caries‐inducing solution comprising 2.2 mM calcium chloride (CaCl_2_), 2.2 mM monosodium phosphate (NaH_2_PO_4_), 0.05 M acetic acid (CH_3_COOH), and adjusted pH to 4.5 with 1 M potassium hydroxide (KOH) using a digital pH meter (Fisher Scientific, Pittsburgh, Pennsylvania, United States) [13]. The tooth specimens were immersed in a caries‐inducing solution and preserved in a humid atmosphere at 37°C for 96 h and then cleaned with DI water to produce a consistent demineralized subsurface enamel lesion.

### 2.3. Evaluation of the Depth of Artificial Demineralized Enamel Lesion

Three demineralized tooth samples were randomly selected for evaluation of the depth of artificial demineralized enamel. Each tooth sample was vertically segmented for a thickness of 200 *μ* with a precision sectioning apparatus (Mecatome T180, PRESI, Eybens, France) and polished with silicon carbide (SiC) abrasive paper up to grit‐5000 and cleaned with DI before microscopically evaluating the polarized light microscopy (PLM, Eclipse‐80i, Nikon, Kanagawa, Japan) at 20× magnification to assess the depth of demineralized enamel.

### 2.4. Application of Infiltration Materials and Challenges in pH‐Cycling

The samples were randomly allocated into three groups (*n* = 20/group) to be infiltrated with different tested materials (Table 1), followed by an acid‐challenging process of pH‐cycling circumstances.

• Group 2R: infiltrated with bioactive remineralized resin infiltration (2R‐infiltration, Lot. No. 2503002018, Dent‐Sci KKU, Khon Kaen, Thailand).
• Group CR: infiltrated with CR infiltration (CR‐infiltration, ICON, Lot. No. 305744, DMG, Hamburg, Germany).
• Group NT: conserved in artificial saliva (AS) to serve as a control group.

**Table 1:** Materials, company, and compositions of materials and solutions used in this study.

Materials	Company	Composition
Bioactive remineralized resin infiltration (2R‐infiltration)	Dent‐Sci KKU, Khon Kaen, Thailand	Triethylene glycol dimethacrylate and ethoxylated bisphenol A dimethacrylate 78%–85%wt, camphorquinone and ethyl 4‐dimethylamino benzoate 1.2%–1.35%wt, nano‐hydroxyapatite and Apacider < 20%wt Lot No.: 25030022018
Conventional resin infiltration (CR‐infiltration)	ICON, DMG, Hamburg, Germany	Triethylene glycol dimethacrylate 70%–95%wt, camphorquinone < 2.5%wt, additional (according to the manufacturer) Lot No.: 305744
Artificial caries inducer (CI)	Biomaterial research, KKU Khon Kaen, Thailand	Calcium chloride 2.2 mM, sodium dihydrogenphosphate 2.2 mM, acetic acid 50 mM, pH adjusted to 5.0 with 1 M potassium hydroxide
Demineralizing solution (DS)	Biomaterial research, KKU Khon Kaen, Thailand	Calcium chloride 1.5 mM, potassium hydrogenphosphate 0.9 mM, acetic acid 50 mM, pH adjusted to 5.0 with 1 M potassium hydroxide
Remineralizing solution (RS)	Biomaterial research, KKU Khon Kaen, Thailand	Calcium chloride 1.5 mM, potassium dihydrogenphosphate 0.9 mM, potassium chloride 130 mM, Hepes 20 mM, pH adjusted to 7.0 with 1 M sodium hydroxide
Artificial saliva (AS)	Biomaterial research, KKU Khon Kaen, Thailand	Potassium chloride 0.65 g/L, magnesium chloride 0.058 g/L, calcium chloride 0.165 g/L, dipotassium hydrogen phosphate 0.804 g/L, potassium dihydrogen phosphate 0.365 g/L, sodium benzoate 2.0 g/L, sodium carboxymethyl cellulose 7.8 g/L, deionized water to make 1 L, pH adjusted to 7.0 with 1 M potassium hydroxide

The demineralized enamel surface of all samples was treated as follows: cleaned with DI water, dried with air spray, applied with 15% HCL (Icon‐etch, DMG) for 2 min, cleaned off with DI water for 30 s, dried with air spray, and then applied with 99% ethanol (Icon‐dry, DMG) for 30 s and left to air‐dry. Group 2R was applied with 2R infiltration on the demineralized surface for two coats. The first coat of 2R‐infiltration was applied with an applicator tip, left for infiltration for 3 min, and then polymerized with a visible light polymerizing unit (Elipar‐S10, 3 M, St. Paul, Minnesota, United States) for 40 s at a distance of 2 mm away from the surface of the material, having a light curing intensity of 800 mW/cm^2^ with a 450 nm wavelength. Afterwards, a second coat of 2R infiltration was applied similarly, left for 60 s, and then polymerized with a visible light polymerizing unit (Elipar‐S10, 3 M) for 40 s at 2 mm from the surface of the material. Group CR was applied with CR infiltration for two coats. The methods for CR application and curing were performed similarly to 2R infiltration, whereas the samples in the NT Group were not treated and stored in the AS. All samples were kept in AS for 24 h, then rinsed with DI water for 1 min before being challenged in the pH‐cycling model for 10 days [14, 15].

The pH‐cycling process comprises the immersion of samples in the demineralized solution (DS), remineralized solution (RS), and AS [16, 17]. The DS composition was 1.5 mM CaCl_2_, 0.9 mM KH_2_PO_4_, 50 mM CH_3_COOH, and adjusted pH to 5.0 [16]. The RS composition was 1.5 mM CaCl_2_, 0.9 mM KH_2_PO_4_, 130 mM KCl, 20 mM Hepes, and adjusted pH to 7.0 [15]. The AS composition was 0.65 g/L KCl, 0.058 g/L MgCl_2_, 0.165 g/L CaCl_2_, 0.804 g/L K_2_HPO_4_, 0.365 g/L KH_2_PO_4_, 2 g/L C_6_H_5_COONa, 7.8 g/L sodium carboxymethyl cellulose, and DI water to make 1 L [18]. The solution was freshly prepared for each cycle. The samples were employed in the pH‐cycling process for 10 days in the shaking apparatus (Wise‐Bath, Seoul, Korea) at 37°C. The pH‐cycling encompassed 0.5 h in DS and 2.5 h in RS 3 times a day, then immersion in AS for 15 h (Figure 1B). The samples were washed with DI water after each transfer to each solution. Group NT was rinsed with DI water 3 times a day and then preserved in the AS for 10 days.

### 2.5. Evaluation of Surface Hardness

**Figure 2:**
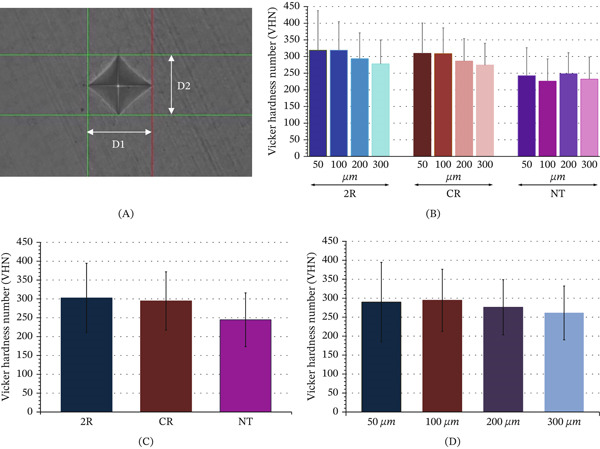
(A) The indentation was measured for diagonal length (D1, D2) and calculated for Vickers hardness number (VHN). (B) Mean and standard deviation of hardness of the artificial demineralized enamel at the level of 50, 100, 200, and 300 *μ*m upon treatment with remineralizing resin (2R), conventional resin (CR) infiltration, and no treatment (NT) group. Differences in mean VHN of demineralized enamel (C) upon treatment with 2R, CR, and NT groups, and (D) at the level of 50, 100, 200, and 300 *μ*m were indicated.

After completion of acid challenging in the pH‐cycling model, all samples were horizontally sectioned at the CEJ using a precision sectioning machine (Mecatome‐T180, Presi). The coronal portions were then embedded in the resin block (width–length–height = 2–2–2 cm) using auto‐polymerized clear acrylic resin. Each sample was then vertically sectioned through the center of the coronal portion in the bucco‐lingual and mesio‐distal direction, resulting in four coronal sections, with eight internal surfaces for each tooth (Figure 1(C4)). The internal surface of each sample was polished with SiC abrasive paper up to grit 5000 and cleansed with DI water. The microhardness was assessed within the tested region of enamel at the levels of 50, 100, 200, and 300 *μ*m from the external surface of enamel. Five indentations were evaluated for each level, with a distance of 100 *μ*m between each indentation (Figure 1(C5)). The 100‐g load, with 15 s dwelling time, was commenced through the Vickers diamond indenter of the microhardness testing apparatus (Future‐tech, Tokyo, Japan) (Figure 1D). The microhardness tester was calibrated periodically throughout the experiment using a standard reference block to ensure consistency. The diagonal distances (D1, D2) of the indented depression were measured (Figure 2A) and calculated for the Vickers hardness number (VHN).

### 2.6. Microscopic Determination

Three samples from each group were vertically segmented for a thickness of 200 *μ*m using a precision sectioning machine (Mecatome T180, PRESI) and polished with SiC abrasive paper up to grit‐5000, cleaned with DI water, and then microscopically examined for PLM with a polarized light microscope (Eclipse‐80i, Nikon) at 10× magnification to assess for the capability of resin infiltration of each group in comparison with the PLM from an ordinary undamaged enamel and demineralized enamel, which were utilized as a reference in assessment.

Three samples from each group were randomly selected and cleansed with distilled water in an ultrasonic cleaner (Vitasonic II, Vita Zahnfabrik, Germany). The specimens were coated with gold–palladium using a sputter coater (K500X, Emitech, Ashford, United Kingdom) at 10 mA current and a 130‐mTorr vacuum for 10 min. Surface morphology was then characterized via scanning electron microscopy (SEM, S‐3000 N, Hitachi, Tokyo, Japan) in secondary electron mode, utilizing an accelerating voltage of 5 kV and a working distance of 7 mm. Micrographs were obtained at 1000 magnification and compared with both sound (undamaged) and demineralized enamel surfaces. The interface between tooth and infiltration materials was examined for the micro‐invasive penetration of resin tags for each infiltrated material. The elemental composition was characterized using energy dispersive spectroscopy (EDS; Oxford, High Wycombe, United Kingdom). For each group (*n* = 3), line scans consisting of 500 points were performed at four distinct levels on each specimen to determine the weight percentages (wt%) of calcium (Ca), phosphorus (P), and oxygen (O).

### 2.7. Statistical Analysis

The data were analyzed for verification of normality with the Kolmogorov–Smirnov test using a statistics software program (SPSS V–28, IBM, Armonk, New York, United States). An analysis of variance (ANOVA) was employed to confirm significant differences in VHN and Ca/P ratio at different levels upon different infiltration materials. Post hoc Fisher′s least significant difference (Fisher′s LSD) multiple comparisons were used to validate the statistically significant differences of VHN and Ca/P ratio among groups at a 95% level of confidence, whereas SEM and PLM were qualitatively evaluated for remineralization potential and infiltration capability of infiltrated resins.

## 3. Results

**Figure 3:**
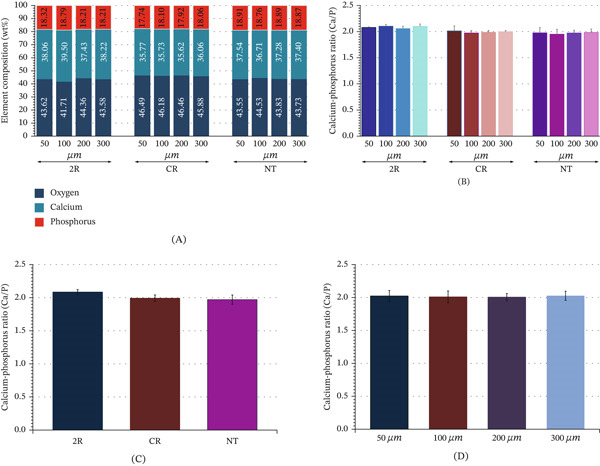
Mean and standard deviation (SD) of (A) elemental compositions in weight percentage (wt.%) of calcium (Ca), phosphorus (P), and oxygen (O) and (B) calcium‐phosphorus ratio (Ca/P) for demineralized enamel at the level of 50, 100, 200, and 300 *μ*m upon treatment with remineralizing resin (2R), conventional resin (CR) infiltration, and no treatment (NT) group. Differences in the mean Ca/P ratio of demineralized enamel (C) upon treatment with 2R, CR, and NT groups, and (D) at the levels of 50, 100, 200, and 300 *μ*m were indicated.

The mean and SD of VHN, elemental composition in wt% of Ca, P, and O, and Ca/P ratio of 2R, CR, and NT groups upon 50, 100, 200, and 300 *μ*m levels of infiltration are displayed (Table 2, Figure 2B, and Figure 3A,B). Two‐way ANOVA was executed to determine whether the type of infiltration material (2R vs. CR) and the level of material infiltration had a significant influence on the microhardness of artificial demineralized enamel in comparison to NT. The statistics revealed that types of infiltration material, level of infiltration, and the combination of material type and level of infiltration had a significant effect on the microhardness of artificial demineralized enamel (*p* < 0.05) (Tables 3, 4, and 5). Post hoc comparisons indicated that artificial demineralized enamel that received 2R infiltration had significantly higher hardness than artificial demineralized enamel that received CR infiltration and NT groups, respectively (*p* < 0.05) (Table 6 and Figure 2C). The multiple comparisons also indicated that the hardness of the infiltrated demineralized enamel at the level of 50 and 100 *μ*m was significantly higher than at the level of 200 and 300 *μ*m, correspondingly (*p* < 0.05). Nevertheless, no significantly different hardness of the infiltrated demineralized enamel at the levels of 50 and 100 *μ*m was indicated (*p* > 0.05) (Table 7 and Figure 2B). The multiple comparisons for the combination of type of infiltration material and level of infiltration indicated no significant difference in the hardness of 2R and CR infiltration, either at the level of 50 or 100 *μ*m (*p* > 0.05), but both were significantly harder than NT for every level (*p* < 0.05). The 2R infiltrated demineralized enamel, either at the level of 50 or 100 *μ*m, indicated significantly higher hardness than the CR infiltrated demineralized enamel, either at the level of 200 *μ* or 300 *μ*m, and also higher than NT for every level (*p* < 0.05). The 2R infiltrated demineralized enamel at the level of 200 *μ*m indicated significantly higher hardness than CR infiltrated demineralized enamel at the level of 300 *μ*m and higher than NT for every level (*p* < 0.05). Nonetheless, the multiple comparisons showed no significant difference between the 2R‐200/CR‐200 and 2R‐300/CR‐200/CR‐300 groups (*p* > 0.05) (Table 8 and Figure 2B).

**Table 2:** Mean, standard deviation (sd) of Vickers hardness number (VHN), chemical element composition [calcium (Ca), phosphorus (P), and oxygen (O)] in weight percentage (wt.%), and Ca/P ratio of demineralized enamel at the level of 50, 100, 200, and 300 *μ*m from the external surface upon treatment with remineralizing resin (2R), conventional resin (CR) infiltration, and no treatment (NT) group.

Group abbreviation	Infiltration material	Level	VHN	Element composition (wt%) (*m* *e* *a* *n* ± *s* *d*)			Ca/P ratio
		*μ*m	*M* *e* *a* *n* ± *s* *d*	Ca	*p*	O	*M* *e* *a* *n* ± *s* *d*
2R‐50	2R	50	318.72 ± 119.18	38.06 ± 1.55	18.32 ± 0.85	43.62 ± 2.39	2.08 ± 0.01
2R‐100	2R	100	318.77 ± 85.68	39.50 ± 0.34	18.79 ± 0.38	41.71 ± 0.67	2.10 ± 0.03
2R‐200	2R	200	293.45 ± 78.51	37.43 ± 1.21	18.21 ± 0.30	44.36 ± 1.46	2.06 ± 0.04
2R‐300	2R	300	277.77 ± 71.74	38.22 ± 0.42	18.21 ± 0.40	43.58 ± 0.67	2.10 ± 0.04
CR‐50	CR	50	309.45 ± 91.14	35.77 ± 3.62	17.74 ± 1.05	46.49 ± 4.65	2.01 ± 0.09
CR‐100	CR	100	308.50 ± 77.59	35.73 ± 1.83	18.10 ± 0.69	46.18 ± 2.45	1.97 ± 0.04
CR‐200	CR	200	286.33 ± 67.56	35.62 ± 1.88	17.92 ± 0.67	46.46 ± 2.54	1.98 ± 0.03
CR‐300	CR	300	274.35 ± 65.29	36.06 ± 1.92	18.06 ± 0.75	45.88 ± 2.65	1.99 ± 0.02
NT‐50	NT	50	242.26 ± 84.24	37.54 ± 5.77	18.91 ± 2.01	43.55 ± 7.77	1.98 ± 0.10
NT‐100	NT	100	255.91 ± 67.12	36.71 ± 5.32	18.76 ± 1.85	44.53 ± 7.15	1.95 ± 0.09
NT‐200	NT	200	248.19 ± 63.15	37.28 ± 3.07	18.89 ± 1.14	43.83 ± 4.18	1.97 ± 0.05
NT‐300	NT	300	232.01 ± 66.69	37.40 ± 2.58	18.87 ± 1.34	43.73 ± 3.48	1.98 ± 0.06

**Table 3:** An analysis of variance (ANOVA) of Vickers microhardness (VHN) of demineralized enamel at the level of 50, 100, 200, and 300 *μ*m from the external surface upon treatment with remineralizing resin (2R), conventional resin (CR) infiltration, and no treatment (NT) group.

Two‐way ANOVA of Vickers microhardness of different materials at different levels					
Source	SS	df	MS	*F*	*p*
Corrected model	2540722.612	11	230974.783	36.547	0.001
Intercept	234147215.657	1	234147215.657	37048.698	0.001
Materials	1943959.461	2	971979.731	153.795	0.001
Level	496622.612	3	165540.871	26.193	0.001
Materials ∗ level	99073.080	6	16512.180	2.613	0.016
Error	18745075.569	2966	6319.985		
Total	255656786.167	2978			
Corrected total	21285798.181	2977			

Abbreviations: df, degree of freedom; F, F‐ratio; MS, mean square; *p*, *p* value; SS, sum of squares.

Element composition (wt.%) for all groups upon detection with EDS comprised Ca, P, and O as principal elements. The composition of Ca was exhibited the highest in 2R infiltrated demineralized enamel compared with the CR and NT groups, whereas the P and O appear to be stable in composition for all groups (Table 2 and Figure 3A). The Ca/P ratio for 2R infiltrated groups was higher than CR and NT groups in every level of demineralized enamel (Table 2 and Figure 3B). Two‐way ANOVA was executed to determine whether the type of material and level of material infiltration had a substantial effect on the ratio of Ca/P in comparison with NT. The statistics revealed that types of infiltration material showed a significant effect on the ratio of Ca/P of artificial demineralized enamel (*p* < 0.05), but no significant difference was observed on the level of infiltration and the combination of material type and level of permeation (*p* > 0.05) (Table 4). Post hoc indicated that artificial demineralized enamel that received 2R infiltration had a significantly higher Ca/P ratio than artificial demineralized enamel that received CR infiltration and NT, respectively (*p* < 0.05) (Table 9 and Figure 3C). Likewise, post hoc confirmed that there was no significant difference in the Ca/P ratio among levels of infiltration (*p* > 0.05) (Table 10 and Figure 3D). Further statistical analysis for the combination of material type and level of infiltration using one‐way ANOVA indicated a significant difference of Ca/P ratio among groups (*p* < 0.05) (Table 5). Remarkably, the post hoc comparisons indicated that the Ca/P of 2R infiltrated demineralized enamel was significantly higher than all NT groups at the levels 50, 100, and 300 *μ*m, except for the level 200 *μ*m (Tables 11 and Figure 3B). Likewise, no significant difference was observed for Ca/P ratio among all groups of the demineralized enamel infiltrated with CR and NT for all levels of infiltration (*p* > 0.05) (Tables 11 and Figure 3B). Contrariwise, the Ca/P ratio for the demineralized enamel infiltrated with 2R at every level of infiltration was significantly higher than the CR‐infiltrated demineralized enamel at the level of 100 *μ*m (*p* < 0.05). In addition, the Ca/P ratio for the demineralized enamel infiltrated with 2R at the level of 50 *μ*m was significantly higher than Ca/P ratio of the CR‐infiltrated demineralized enamel at the level of 100 *μ*m (*p* < 0.05), but there was no significant difference with CR infiltration at the level of 50 *μ*m (*p* > 0.05) (Tables 11 and Figure 3B). Interestingly, no significant difference was observed for Ca/P ratio of demineralized enamel infiltrated with 2R and CR infiltration at levels 50, 200, and 300 *μ*m.

**Table 4:** An analysis of variance (ANOVA) of calcium‐phosphorus ratio (Ca/P) of demineralized enamel at the level of 50, 100, 200, and 300 *μ*m from the external surface upon treatment with remineralizing resin (2R), conventional resin (CR) infiltration, and no treatment (NT) group.

Two‐way ANOVA of Ca/P ratio of different materials at different levels					
Source	SS	df	MS	*F*	*p*
Corrected model	0.097	11	0.009	2.364	0.038
Intercept	146.329	1	146.329	39283.045	0.001
Materials	0.088	2	0.044	11.844	0.001
Level	0.003	3	0.001	0.263	0.851
Materials ∗ level	0.006	6	0.001	0.254	0.953
Error	0.089	24	0.004		
Total	146.516	36			
Corrected total	0.186	35			

Abbreviations: df, degree of freedom; F, F‐ratio; MS, mean square; *p*, *p* value; SS, sum of squares.

**Table 5:** An analysis of variance (ANOVA) of calcium‐phosphorus ratio (Ca/P) of demineralized enamel at the level of 50, 100, 200, and 300 *μ*m from the external surface upon treatment with remineralizing resin (2R), conventional resin (CR) infiltration, and no treatment (NT) group.

One‐way ANOVA of Ca/P ratio among groups of material at each level					
Source	SS	df	MS	*F*	*p*
Corrected model	0.097	11	0.009	2.364	0.038
Intercept	146.329	1	146.329	39283.045	0.001
Groups	0.097	11	0.009	2.364	0.038
Error	0.089	24	0.004		
Total	146.516	36			
Corrected total	0.186	35			

Abbreviations: df, degree of freedom; F, F‐ratio; MS, mean square; *p*, *p* value; SS, sum of squares.

**Table 6:** Post hoc Fisher′s least significant difference (Fisher′s LSD) multiple comparisons of Vickers hardness of demineralized enamel upon treatment with remineralized resin (2R), conventional resin (IC) infiltration, and no treatment (NT).

Fisher′s LSD multiple comparison of Vickers hardness upon different treated materials			
Material	2R	CR	NT
2R	1.000	0.028	0.001
CR		1.000	0.001
NT			1.000
			

**Table 7:** Post hoc Fisher′s least significant difference (Fisher′s LSD) multiple comparisons of Vickers hardness of demineralized enamel at the level of 50, 100, 200, and 300 *μ*m from the external surface.

Fisher′s LSD multiple comparison of Vickers hardness upon different levels of infiltration				
Level	50 *μ*m	100 *μ*m	200 *μ*m	300 *μ*m
50 *μ*m	1.000	0.246	0.001	0.001
100 *μ*m		1.000	0.001	0.001
200 *μ*m			1.000	0.001
300 *μ*m				1.000

**Table 8:** Post hoc Fisher′s least significant difference (Fisher′s LSD) multiple comparisons of Vickers hardness of demineralized enamel upon the combination of material (Mat) and level of infiltration (Lev).

Fisher′s LSD multiple comparison of Vickers hardness upon the combination of materials (Mat) and level of infiltration (Lev)												
Mat ∗ Lev	2R‐50	2R‐100	2R‐200	2R‐300	CR‐50	CR‐100	CR‐200	CR‐300	NT‐50	NT‐100	NT‐200	NT‐300
2R‐50	1.000	0.994	0.001	0.001	0.199	0.152	0.001	0.001	0.001	0.001	0.001	0.001
2R‐100		1.000	0.001	0.001	0.192	0.145	0.001	0.001	0.001	0.001	0.001	0.001
2R‐200			1.000	0.029	0.025	0.033	0.314	0.007	0.001	0.001	0.001	0.001
2R‐300				1.000	0.001	0.001	0.235	0.636	0.001	0.002	0.001	0.001
CR‐50					1	0.893	0.001	0.001	0.001	0.001	0.001	0.001
CR‐100						1.000	0.002	0.001	0.001	0.001	0.001	0.002
CR‐200							1.000	0.092	0.001	0.001	0.001	0.001
CR‐300								1.000	0.001	0.009	0.001	0.001
NT‐50									1.000	0.055	0.406	0.152
NT‐100										1.000	0.277	0.001
NT‐200											1.000	0.024
NT‐300												1.000

**Table 9:** Post hoc Fisher′s least significant difference (Fisher′s LSD) multiple comparisons of calcium‐phosphorus (Ca/P) ratio of demineralized enamel upon treatment with remineralized resin (2R), conventional resin (CR) infiltration, and no treatment (NT).

Fisher′s LSD multiple comparison of Ca/P ratio upon different treated materials			
Material	2R	CR	NT
2R	1.000	0.001	0.001
CR		1.000	0.393
NT			1.000
			

**Table 10:** Post hoc Fisher′s least significant difference (Fisher′s LSD) multiple comparisons of calcium‐phosphorus (Ca/P) ratio of demineralized enamel at the level of 50, 100, 200, and 300 *μ*m from the external surface.

Fisher′s LSD multiple comparison of Ca/P ratio upon different levels of infiltration				
Level	50 *μ*m	100 *μ*m	200 *μ*m	300 *μ*m
50 *μ*m	1.000	0.620	0.542	0.909
100 *μ*m		1.000	0.909	0.542
200 *μ*m			1.000	0.470
300 *μ*m				1.000

**Table 11:** Post hoc Fisher′s least significant difference (Fisher′s LSD) multiple comparisons of calcium‐phosphorus (Ca/P) ratio of demineralized enamel upon the combination of material (Mat) and level of infiltration (Lev).

Fisher′s LSD multiple comparison of Ca/P ratio upon the combination of materials (Mat) and level of infiltration (Lev)												
Mat ∗ Lev	2R‐50	2R‐100	2R‐200	2R‐300	CR‐50	CR‐100	CR‐200	CR‐300	NT‐50	NT‐100	NT‐200	NT‐300
2R‐50	1.000	0.644	0.644	0.692	0.194	0.043	0.073	0.107	0.049	0.015	0.043	0.064
2R‐100		1.000	0.358	0.947	0.083	0.015	0.028	0.043	0.018	0.005	0.015	0.024
2R‐200			1.000	0.393	0.393	0.107	0.173	0.240	0.121	0.043	0.107	0.151
2R‐300				1.000	0.095	0.018	0.032	0.049	0.021	0.006	0.018	0.028
CR‐50					1.000	0.430	0.597	0.741	0.469	0.216	0.430	0.553
CR‐100						1.000	0.791	0.644	0.947	0.644	1.000	0.843
CR‐200							1.000	0.843	0.843	0.469	0.791	0.947
CR‐300								1.000	0.692	0.358	0.644	0.791
NT‐50									1.000	0.597	0.947	0.895
NT‐100										1.000	0.644	0.510
NT‐200											1.000	0.843
NT‐300												1.000

**Figure 4:**
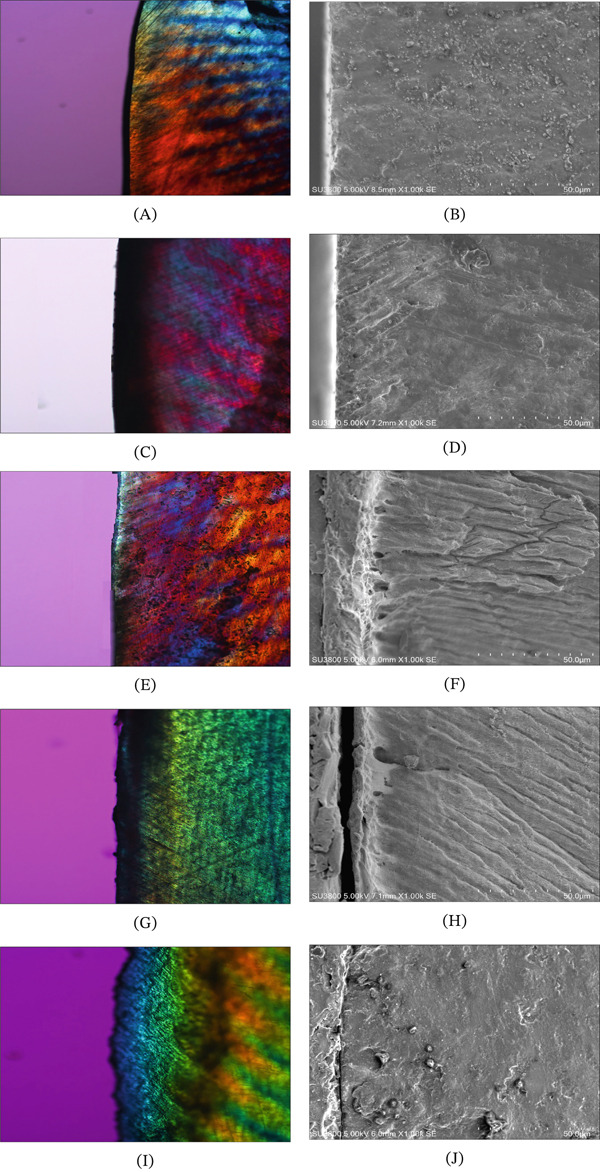
(A, C, E, G, I) Polarized light microscopies (PLM) at ×10 and (B, D, F, H, J) scanning electron microscopies (SEM) at ×1000 magnification of (A, B) undamaged enamel, (C, D) demineralized enamel, demineralized enamel after application of pH‐cycling and treatment with (E, F) remineralizing resin (2R) infiltration, (G, H) conventional resin (CR) infiltration, and (I, J) no treatment (NT) group.

PLM findings of the appearance of a demineralized enamel lesion together with the advancement of the remineralization procedure upon treatment with 2R, CR, and NT groups (Figure 4E, G, and I) were compared with the PLM of normal intact enamel (Figure 4A) and demineralized enamel (Figure 4C). A noticeable body of lesion and intensifying lesion depth were noticed from the PLM of the artificial demineralized enamel lesion, for approximately ranged 103.7–243.8 *μ*m from the external surface of enamel (Figure 4C). Once the infiltration materials were applied, followed by the pH‐cycling processes, the lessening depth of lesion for each infiltration material was indicated (Figure 4E,G), except for NT group that denoted an upsurge in depth of lesion (Figure 4I) comparing to the early lesion depth (Figure 4C). It advised that each type of infiltration resin was capable of decreasing depth of the artificial demineralized enamel. The diminution in demineralized lesions for 2R infiltration was significantly superior to CR infiltration and the NT groups, correspondingly. Still, the infiltration of 2R into the depth of the synthetic demineralized enamel lesion perhaps indicated a somewhat deeper and greater penetration than the CR infiltration. Enhancement in the lesion depth of demineralized enamel for NT was evident, and remineralization was not observed during the process of pH‐cycles.

SEM findings among groups were identified and contrasted with the normal intact (Figure 4B) and demineralized enamel (Figure 4D) at ×1K magnification. The artificially demineralized enamel showed an irregular pattern of gashed and impaired regions beside severe marks of porosity (Figure 4D) compared with the uniform and intact distinctive enamel configuration (Figure 4B). Several sponginess and disparity were evident of surface loss, classically correlated with unestablished remineralization for NT during the process of pH cycles (Figure 4J). On the contrary, the superior penetration depth of the 2R infiltration material and the mineral contents (white interfacial zone) deeply penetrated to demineralized enamel along the enamel rods, reaching further into the subsurface carious layers, representing the creation of more HA deposit and mineralization progression to the demineralized enamel (Figure 4F). The CR infiltration showed a comparatively shallower penetration of the resin into the demineralized enamel lesion (Figure 4H) compared with the 2R infiltration. The SEM signified a progress of HA remineralization processes founded on the artificial demineralized enamel once treated with 2R, whereas lesser resin infiltration was observed when treated with CR. Contrarily, hidden porosities due to surface loss and an obviously irregular damaging pattern of demineralized enamel were found in the nontreated group (Figure 4J).

## 4. Discussion

Contemporary restorative dentistry considers remineralization as an interceptive approach to incipient carious lesions of enamel. This study evaluated the competencies of 2R and CR infiltration on remineralization of the initial demineralized enamel lesions in comparison to no treatment (NT) upon a pH‐cycling model, based on the determination with VHN, PLM, SEM, and EDS. The study signified that 2R infiltration possessed significantly better remineralization capability in improving demineralized enamel than CR infiltration and NT. Thus, the null hypothesis was rejected. The 2R appears to have better remineralization capability than CR and NT, respectively. The efficacy of remineralization was supported by the microscopic evidence. The calcium and phosphate elements were higher in the demineralized lesion for the 2R than the CR and NT groups. Particularly, higher diminution in lesion depth of demineralized enamel for 2R than CR and NT was supported by PLM.

Progression of remineralization was capable of precise evaluation for comparing the effect of different infiltrated materials using PLM, which were then accurately measured [5, 6, 19, 20]. A thickening dark band beneath the superficial enamel surface indicated demineralization. Although a diminutive of dark bandwidth in 2R infiltrated demineralized enamel, compared with CR and NT, indicated the potential of 2R in remineralization. It indicated that 2R‐infiltrated demineralized enamel offered the highest decrease in depth of demineralized enamel. It is possibly owing to the principal calcium phosphate composition in 2R, in which the small radius of Ca^2+^ (0.99 Å) and PO4^3−^ (1.15 Å) particles offered the nHA superior infiltration, higher accumulation in the skeletons of demineralized enamel, and significantly reduced the depth of the lesion compared with others [5, 6, 21].

Both 2R and CR infiltrations were applied only once until the end of the experiment to simulate. The 2R exhibited higher remineralization of demineralized enamel lesions by providing Ca^2+^ and PO4^3−^ ion diffusion into the subsurface area of the lesion for establishing structural reparations at the molecular level. Upon reaching the subsurface area, Ca^2+^ and PO4^3−^ ions interact with residual hydroxyapatite crystallites and organic matrix components within the demineralized enamel. These ions precipitate to form new mineral deposits, primarily as hydroxyapatite [Ca10(PO4)6(OH)2] or precursor phases, which integrate with existing enamel ultrastructure. This process reconstitutes the prismatic architecture and restores mechanical integrity at the nanoscale level [22, 23]. The role of mineral diffusion is crucially associated with the porosity and depth of demineralized enamel lesions. The larger the porosities, the higher the amount of mineral deposit; yet the deeper the lesions, the longer the penetration distance of penetration, and the more difficulty for mineral diffusion. The 2R infiltration possessed the small molecules of Ca^2+^ and PO4^3−^ ions, which could be diffused into the deeper level of demineralized enamel and accumulated higher Ca/P contents than CR and NT, as reflected by the higher Vickers microhardness. Some studies reported the techniques to enhance resin penetration by removing the hypermineralized pseudo‐intact layer of demineralized enamel surface by etching with 15% HCl before infiltration [22–24]. The etched surface for 120 s has been shown to remove the hypermineralized enamel layer to a depth of approximately 36.70 ± 7.62 *μ*m [24]. This can expose enamel porosities and disrupt the prismatic layer, thereby enhancing resin penetration. Therefore, in the present study, every demineralized enamel surface was prepared in the same manner before infiltration with resins.

The study showed that both resin infiltration materials increased microhardness in artificially demineralized enamel lesions, with 2R groups showing higher hardness values than CR groups for every depth. The superior performance of 2R at those depths may be attributed to the presence of nHA with tricalcium phosphate and nano‐silica from Apacider, which is known to facilitate mineral deposition and enhance enamel surface hardness. Both the 2R and CR groups showed significantly greater microhardness than the NT group at all depths. These results suggest that etching followed by infiltration enhances resin penetration and reinforces the hardness of the enamel surface, consistent with a previous report that infiltration resins efficiently help improve enamel with white spot lesions [25, 26]. Resin infiltration exhibits a profound capacity to permeate almost entirely the enamel substance of naturally occurring carious lesions. This study indicated that 2R was capable of enhancing remineralization, which was also consistent with the microscopic evidence.

The calcium/phosphorus (C/P) ratio was analyzed using SEM and EDS, highlighting a trend of Ca deposition for specimens exposed to 2R infiltration. The analysis of the C/P ratio revealed notable distinctions among different groups. The 2R exhibited a greater Ca/P ratio than both the CR and NT groups, suggesting a more pronounced effect of the 2R infiltration material relative to CR infiltration. It is also important to consider the etching protocols that involve an initial acidic etching step, typically with 15% HCl, which is crucial for removing the superficial enamel layer and creating micro‐porosities to facilitate resin penetration [5]. This procedure, however, inherently leads to some degree of loss from the enamel surface due to demineralization [5]. Accordingly, the Ca/P ratio value for CR infiltration in comparison to the NT group indicated that CR resulted in a comparable Ca/P ratio to the NT. This suggested that the acidic challenging process without sufficient compensatory remineralization to overcome initial demineralization could contribute to a decreased Ca/P ratio, as validated in the CR group. However, despite undergoing a similar acidic challenging process, the 2R group consistently demonstrated a higher Ca/P ratio than both the CR and NT groups. This divergent outcome strongly suggests that the composition of the 2R infiltration material plays a critical role. Unlike CR infiltration, the 2R infiltration incorporates various components such as nHA and Apacider that contain silver, zinc, silica, and tricalcium phosphate [5, 6, 12]. These active ingredients probably contribute to a net positive calcium balance, either by facilitating remineralization or by providing a robust protective barrier that mitigates further demineralization more effectively than the conventional infiltration, thereby offsetting the Ca loss induced by the acidic challenging step and ultimately resulting in an augmented Ca/P ratio.

The competence of ion release of bioactive particles contained in resin materials is generally related to the amount and surface area of the particles, the hydrophilic properties of the resin, and the acid‐base condition of the environment [21, 27]. This study concurred with earlier reports that exhibited increasing calcium and phosphate release from 2R infiltrations incorporating nHA and tricalcium phosphate fillers once the pH of the immersion solution is reduced [21, 27]. The study displayed the greater Ca release observed from 2R infiltrations compared with CR infiltration, probably attributable to the nanoscale dimensions of the nHA particles in the 2R formulation. Therefore, 2R formulations containing nHA and tricalcium phosphate from Apacider are likely to function as effective remineralizing agents under cariogenic conditions. However, a high Ca/P ratio only indicates the relative abundance of elements and does not necessarily confirm the stable incorporation within the hydroxyapatite molecular structure. To ascertain the precise hydroxyapatite crystals, an x‐ray diffraction analysis would be necessary, which will be subsequently reported in our research thereafter.

The quest for depth upon resin penetration is a critical determinant of diffusion barrier formation and potentiates the effectiveness of infiltration in arresting caries progression, as concluded from a previous study [28]. This study appraised the intensity of material penetration using SEM and EDS and disclosed that 2R exhibited a superior infiltration depth compared with CR. The enhanced penetration capability in 2R can be attributed to the capillary action of the nano‐sized nHA particles (< 60 nm) through the wetting surface of interprismatic spaces, along with a low‐viscosity medium carrier, which increases the penetration coefficient as described by the Washburn equation and promotes capillary‐driven flow into the lateral surface of enamel rods [28]. The SEM microscopies confirmed the penetration of nHA particles into the depths of demineralized enamel lesions and formed a homogenous apatite layer, since the tinier size of the particle and the good dynamic of 2R feasibly promote penetration into subsurface lesions and continually seal the sponginess of the demineralized enamel. This finding is consistent with previous reports indicating that nanotechnology is a promising strategy for producing materials with improved remineralization potential [6, 10, 25, 29]. With the nanosized particles, the bioactivity of calcium‐ and phosphate‐based fillers increases and enhances the ability to infiltrate porosities within demineralized regions to act as effective remineralizing agents [10, 30].

The SEM and EDS were used to assess qualitative alterations at the tooth surface–resin interface; the presence of a white interfacial zone was interpreted as evidence of remineralization [29]. A homogeneous, white remineralized layer at the enamel–resin interface was detected in 2R infiltration comprising nHA and tricalcium phosphate from Apacider with greater prominence compared with the IC group. The CR‐treated specimens showed a consistent smoothness at the tooth interface in limited areas. This evidence was consistent with several previous reports [29–31]. Park et al. noticed an increasingly distinct white granular zone with higher hydroxyapatite content [28]. Roza et al. observed that nHA precipitates′ thickness layer correlated with HAP concentration [30], whereas Choudhary et al. reported a white zone at the tooth–resin interface when evaluating remineralization by the products containing fluoride and amorphous calcium phosphate [31]. The microscopic investigation from this study aligned consistently with the result of microhardness measurements, where 2R infiltration established significantly higher microhardness values, indicating higher remineralization potential than CR and NT.

The study is still limited to the simulation of bacterial biofilm and salivary pellicle in the oral cavity. Moreover, the conceivable dissimilarities of each tooth, from different ages of donors and diverse experiences to oral biota, led to the aberration in responses to acid‐challenging conditions. Likewise, the demineralization‐remineralization process was shorter than in natural conditions. Therefore, an extrapolation of the research to clinical circumstances is still limited. Several multifactorial factors, including simulated salivary components and flow, pellicle and biofilm formation, masticatory loading, and intraoral thermal fluctuations—conditions that probably affect polymerization shrinkage and hygroscopic expansion, as well as long‐term material behavior—should be considered for further investigation. Likewise, the immersion‐based acid demineralization protocol employed to create carious lesions might not apprehend the heterogeneous and dynamic carious microstructure of enamel that develops in vivo. Accordingly, confirmation in situ or clinical studies is necessary. Further scientific studies are recommended for clinical studies. Nevertheless, the clinical implication of this study is crucial in leading the investigated 2R infiltration material to convey praiseworthy information for dentists to pursue in professional practice.

## 5. Conclusion

This in vitro study investigated the effectiveness of the 2R infiltration on remineralization of the demineralized enamel lesion. Although both remineralizing resin (2R) and CR improve the microhardness of demineralized enamel, 2R exhibits significantly greater remineralization potential. Due to its deeper penetration and superior calcium replenishment compared with CR, 2R is a more effective minimal intervention material for early demineralized enamel lesions. However, clinical trials are necessary to validate these findings in vivo.

## Author Contributions

Niwut Juntavee, Apa Juntavee, and Kemporn Kitsahawong conceived the concept and design, analyzed the data, supervised the project, and prepared the manuscript. Chalita Suwannasuk, Chayapha Siriwatthanamethanon, Nattanicha Buddeesee, and Rachata Somsub carried out the experiment, data collection, and analysis.

## Funding

This study was supported by Fundamental Fund 2568, Thailand Science Research and Innovation, Ministry of Higher Education, Science, Research and Innovation, Royal Thai Government (4777602).

## Disclosure

The authors have nothing to report.

## Conflicts of Interest

The authors declare no conflicts of interest.

## General Statement

*Clinical Significance*. Modern dentistry has been targeting minimally invasive treatment through the use of materials for the remineralization procedure. 2R is a bioactive resin infiltration material that is extremely capable of penetrating and enhancing the hardness of demineralized enamel more than conventional resin infiltration. Thus, 2R can be recommended as a potential remineralization material for handling initial carious lesions in dental practice.

## Data Availability

The data that support the findings of this study are available from the corresponding author upon reasonable request.

## References

[1] Lin G. S. S. , Chan D. Z. K. , Lee H. Y. , Low T. T. , Laer T. S. , Pillai M. P. , Yew Y. Q. , Saadun S. W. , and Wafa T. , Effectiveness of Resin Infiltration in Caries Inhibition and Aesthetic Appearance Improvement of White-Spot Lesions: An Umbrella Review, Journal of Evidence-Based Dental Practice. (2022) 22, no. 3, 101723, 10.1016/j.jebdp.2022.101723, 36162890. 36162890

[2] Bourouni S. , Dritsas K. , Kloukos D. , and Wierichs R. J. , Efficacy of Resin Infiltration to Mask Post-Orthodontic or Non-Post-Orthodontic White Spot Lesions or Fluorosis—A Systematic Review and Meta-Analysis, Clinical Oral Investigations. (2021) 25, no. 8, 4711–4719, 10.1007/s00784-021-03931-7, 34106348. 34106348 PMC8342329

[3] Cebula M. , Göstemeyer G. , Krois J. , Pitchika V. , Paris S. , Schwendicke F. , and Effenberger S. , Resin Infiltration of Non-Cavitated Proximal Caries Lesions in Primary and Permanent Teeth: A Systematic Review and Scenario Analysis of Randomized Controlled Trials, Journal of Clinical Medicine. (2023) 12, no. 2, 10.3390/jcm12020727, 36675656. PMC9864315 36675656

[4] El-Meligy O. , Alamoudi N. M. , Eldin-Ibrahim S. T. , Felemban O. M. , and Al-Tuwirqi A. A. , Effect of Resin Infiltration Application on Early Proximal Caries Lesions in vitro, Journal of Dental Sciences. (2021) 16, no. 1, 296–303, 10.1016/j.jds.2020.04.005, 33384812. 33384812 PMC7770447

[5] Juntavee A. , Juntavee N. , Pongpanatnukul C. , Kruemai K. , and Limrachtamorn T. , Remineralization Potential of Apacider Mangosteen Adhesive Pastes on Artificial Carious Lesions, Journal of Dental Sciences. (2024) 19, no. 2, 978–989, 10.1016/j.jds.2023.07.012, 38618135. 38618135 PMC11010799

[6] Juntavee A. , Juntavee N. , and Sinagpulo A. N. , Nano-Hydroxyapatite Gel and Its Effects on Remineralization of Artificial Carious Lesions, International Journal of Dentistry. (2021) 2021, 12, 10.1155/2021/7256056, 34790238. PMC8592696 34790238

[7] O′Hagan-Wong K. , Enax J. , Meyer F. , and Ganss B. , The Use of Hydroxyapatite Toothpaste to Prevent Dental Caries, Odontology. (2022) 110, no. 2, 223–230, 10.1007/s10266-021-00675-4, 34807345. 34807345 PMC8930857

[8] Amaechi B. T. , Alshareif D. O. , Azees P. A. A. , Shehata M. A. , Lima P. P. , Abdollahi A. , Kalkhorani P. S. , Evans V. , Bagheri A. , and Okoye L. O. , Anti-Caries Evaluation of a Nano-Hydroxyapatite Dental Lotion for Use After Toothbrushing: An In Situ Study, Journal of Dentistry. (2021) 115, 103863, 10.1016/j.jdent.2021.103863, 34743963. 34743963

[9] Scribante A. , Dermenaki Farahani M. R. , Marino G. , Matera C. , Rodriguez Y Baena R. , Lanteri V. , and Butera A. , Biomimetic Effect of Nano-Hydroxyapatite in Demineralized Enamel Before Orthodontic Bonding of Brackets and Attachments: Visual, Adhesion Strength, and Hardness in In Vitro Tests, BioMed Research International. (2020) 2020, 6747498, 10.1155/2020/6747498, 32090106. 32090106 PMC7013302

[10] Pawinska M. , Paszynska E. , Limeback H. , Amaechi B. T. , Fabritius H. O. , Ganss B. , O’Hagan-Wong K. , Wiesche E. S. , Meyer F. , and Enax J. , Hydroxyapatite as an Active Ingredient in Oral Care: An International Symposium Report, Bioinspired, Biomimetic and Nanobiomaterials. (2024) 13, no. 1, 1–14, 10.1680/jbibn.23.00034.

[11] Arifa M. K. , Ephraim R. , and Rajamani T. , Recent Advances in Dental Hard Tissue Remineralization: A Review of Literature, International Journal of Clinical Pediatric Dentistry. (2019) 12, no. 2, 139–144, 10.5005/jp-journals-10005-1603, 31571787. 31571787 PMC6749882

[12] Sodata P. , Juntavee A. , Juntavee N. , and Peerapattana J. , Optimization of Adhesive Pastes for Dental Caries Prevention, AAPS PharmSciTech. (2017) 18, no. 8, 3087–3096, 10.1208/s12249-017-0750-0, 2-s2.0-85019573333, 28516412. 28516412

[13] Alagha E. and Alagha M. I. , Comparing Impact of Two Resin Infiltration Systems on Microhardness of Demineralized Human Enamel After Exposure To Acidic Challenge, Open Access Macedonian Journal of Medical Sciences. (2021) 9, no. D, 92–97, 10.3889/oamjms.2021.5878.

[14] Malekafzali B. , Ekrami M. , Mirfasihi A. , and Abdolazimi Z. , Remineralizing Effect of Child Formula Dentifrices on Artificial Enamel Caries Using a pH Cycling Model, Journal of Dentistry. (2015) 12, no. 1, 11–17. 26005449 PMC4436322

[15] Oliveira G. M. , Ritter A. V. , Heymann H. O. , Swift E. , Donovan T. , Brock G. , and Wright T. , Remineralization effect of CPP-ACP and fluoride for white spot lesions in vitro, Journal of Dentistry. (2014) 42, no. 12, 1592–1602, 10.1016/j.jdent.2014.09.004, 2-s2.0-84913532078, 25260438. 25260438 PMC5551488

[16] Al-Asmar A. A. , Mineralization of Dentinal Lesions With Different Concentrations of Fluoride, International Journal of Dentistry. (2024) 2024, no. 1, 3476050, 10.1155/2024/3476050, 38550542. 38550542 PMC10977341

[17] Kumar V. L. , Itthagarun A. , and King N. M. , The Effect of Casein Phosphopeptide-Amorphous Calcium Phosphate on Remineralization of Artificial Caries-Like Lesions: An In Vitro Study, Australian Dental Journal. (2008) 53, no. 1, 34–40, 10.1111/j.1834-7819.2007.00006.x, 2-s2.0-41849147919, 18304239. 18304239

[18] Panich M. and Poolthong S. , The Effect of Casein Phosphopeptide-Amorphous Calcium Phosphate and a Cola Soft Drink on In Vitro Enamel Hardness, Journal of the American Dental Association. (2009) 140, no. 4, 455–460, 10.14219/jada.archive.2009.0195, 2-s2.0-64749115772, 19339535. 19339535

[19] Schwendicke F. , Eggers K. , Meyer-Lueckel H. , Dörfer C. , Kovalev A. , Gorb S. , and Paris S. , In vitro Induction of Residual Caries Lesions in Dentin: Comparative Mineral Loss and Nano-Hardness Analysis, Caries Research. (2015) 49, no. 3, 259–265, 10.1159/000371897, 2-s2.0-84926223832, 25832626. 25832626

[20] Soares R. , De Ataide I. D. N. , Fernandes M. , and Lambor R. , Assessment of Enamel Remineralisation after Treatment With Four Different Remineralising Agents: a Scanning Electron Microscopy (SEM) study, Journal of Clinical and Diagnostic Research. (2017) 11, no. 4, ZC136–ZC141, 10.7860/JCDR/2017/23594.9758, 28571281. 28571281 PMC5449906

[21] Xu H. H. , Moreau J. L. , Sun L. , and Chow L. C. , Nanocomposite Containing Amorphous Calcium Phosphate Nanoparticles for Caries Inhibition, Dental Materials. (2011) 27, no. 8, 762–769, 10.1016/j.dental.2011.03.016, 2-s2.0-79958045075. 21514655 PMC3125490

[22] Paris S. , Soviero V. M. , Schuch M. , and Meyer-Lueckel H. , Pretreatment of Natural Caries Lesions Affects Penetration Depth of Infiltrants In Vitro, Clinical oral Investigations. (2013) 17, no. 9, 2085–2089, 10.1007/s00784-012-0909-8, 2-s2.0-84888638621, 23262835. 23262835

[23] Lausch J. , Paris S. , Selje T. , Dörfer C. E. , and Meyer-Lückel H. , Resin Infiltration of Fissure Caries With Various Techniques of Pretreatment In Vitro, Caries Research. (2015) 49, no. 1, 50–55, 10.1159/000366082, 2-s2.0-84923546965, 25427531. 25427531

[24] López López E. A. , Dominguez J. A. , and Gomes G. M. , Effect of Conditioning Protocols and Ultrasonic Application of An Infiltrant Resin in White Spot Lesions, Brazilian Dental Journal. (2019) 30, no. 1, 58–65, 10.1590/0103-6440201902329, 2-s2.0-85062854644. 30864649

[25] Sahiti J. S. , Krishna N. V. , Prasad S. D. , Kumar C. S. , Kumar S. S. , and Babu K. S. C. , Comparative Evaluation of Enamel Microhardness After Using Two Different Remineralizing Agents on Artificially Demineralized Human Enamel: An In Vitro Study, Journal of Clinical and Translational Research. (2020) 6, no. 3, 87–91, 33426358. 33426358 PMC7790502

[26] Soveral M. , Machado V. , Botelho J. , Mendes J. J. , and Manso C. , Effect of Resin Infiltration on Enamel: A Systematic Review and Meta-Analysis, Journal of Functional Biomaterials. (2021) 12, no. 3, 10.3390/jfb12030048, 34449679. PMC8395859 34449679

[27] Xu H. H. , Weir M. D. , and Sun L. , Calcium and Phosphate Ion Releasing Composite: effect of pH on Release and Mechanical Properties, Dental Materials. (2009) 25, no. 4, 535–542, 10.1016/j.dental.2008.10.009, 2-s2.0-60449096750, 19101026. 19101026 PMC2649691

[28] Ibrahim D. F. A. , Venkiteswaran A. , and Hasmun N. N. , The Penetration Depth of Resin Infiltration Into Enamel: A Systematic Review, Journal of International Society of Preventive & Community Dentistry. (2023) 13, no. 3, 194–207, 10.4103/jispcd.JISPCD_36_23, 37564169. 37564169 PMC10411299

[29] Park S. W. , Lee Y. K. , Kim Y. U. , Kim M. C. , Kim K. N. , Choi B. J. , and Choi H. J. , The Effect of Hydroxyapatite on the Remineralization of Dental Fissure Sealant, Key Engineering Materials. (2005) 284-286, 35–38, 10.4028/www.scientific.net/KEM.284-286.35.

[30] Roza H. , Mohammad A. , Sara Tavassoli H. , Somayeh K. , and Sara Rahimian I. , The Effect of Various Amounts of Nanohydroxyapatite on the Mechanical Properties and Remineralization of a Fissure Sealant, Journal of Dental School-Shahid Beheshti University of Medical Sciences. (2021) 30, no. 3, 184–191, http://www.magiran.com/p1053055.

[31] Choudhary P. , Tandon S. , Ganesh M. , and Mehra A. , Evaluation of the Remineralization Potential of Amorphous Calcium Phosphate and Fluoride-Containing Pit and Fissure Sealants Using Scanning Electron Microscopy, Indian Journal of Dental Research. (2012) 23, no. 2, 157–163, 10.4103/0970-9290.100419, 2-s2.0-84866264495, 22945703. 22945703

